# A 19-isolate reference-quality global pangenome for the fungal wheat pathogen *Zymoseptoria tritici*

**DOI:** 10.1186/s12915-020-0744-3

**Published:** 2020-02-11

**Authors:** Thomas Badet, Ursula Oggenfuss, Leen Abraham, Bruce A. McDonald, Daniel Croll

**Affiliations:** 1 Laboratory of Evolutionary Genetics, Institute of Biology, University of Neuchâtel, Neuchâtel, Switzerland; 2 Plant Pathology, Institute of Integrative Biology, ETH Zürich, Zürich, Switzerland

## Abstract

**Background:**

The gene content of a species largely governs its ecological interactions and adaptive potential. A species is therefore defined by both core genes shared between all individuals and accessory genes segregating presence-absence variation. There is growing evidence that eukaryotes, similar to bacteria, show intra-specific variability in gene content. However, it remains largely unknown how functionally relevant such a pangenome structure is for eukaryotes and what mechanisms underlie the emergence of highly polymorphic genome structures.

**Results:**

Here, we establish a reference-quality pangenome of a fungal pathogen of wheat based on 19 complete genomes from isolates sampled across six continents. *Zymoseptoria tritici* causes substantial worldwide losses to wheat production due to rapidly evolved tolerance to fungicides and evasion of host resistance. We performed transcriptome-assisted annotations of each genome to construct a global pangenome. Major chromosomal rearrangements are segregating within the species and underlie extensive gene presence-absence variation. Conserved orthogroups account for only ~ 60% of the species pangenome. Investigating gene functions, we find that the accessory genome is enriched for pathogenesis-related functions and encodes genes involved in metabolite production, host tissue degradation and manipulation of the immune system. De novo transposon annotation of the 19 complete genomes shows that the highly diverse chromosomal structure is tightly associated with transposable element content. Furthermore, transposable element expansions likely underlie recent genome expansions within the species.

**Conclusions:**

Taken together, our work establishes a highly complex eukaryotic pangenome providing an unprecedented toolbox to study how pangenome structure impacts crop-pathogen interactions.

**Electronic supplementary material:**

The online version of this article (10.1186/s12915-020-0744-3) contains supplementary material, which is available to authorized users.

## Background

Microbial species harbour substantial functional diversity at the level of gene presence-absence variation [1]. Genes not fixed within a species (i.e. accessory genes) can account for a large fraction of the full gene repertoire (i.e. the pangenome). In bacteria, the proportion of core genes in the pangenome can range from 5 to 98% and challenge taxonomic classifications [2, 3]. The wide spectrum of pangenome sizes across species can be associated with the species distribution and lifestyle [4]. Species showing a wide geographical distribution and large population sizes characterized by frequent genetic exchange tend to have expansive, open pangenomes [5]. In microbial pathogens, accessory genes play a major role in virulence and environmental adaptation [6–8]. The notion of a pangenome led to the discovery that major elements of intra-specific variation are often ignored in studies relying on a single reference genome. Large pangenomes also can challenge association studies aiming to identify the genetic basis of phenotypic traits because mapping is often performed against a single reference genome, making potentially relevant genetic variation inaccessible [9, 10]. Despite their importance for unraveling the genetic basis of adaptive evolution, only a very limited number of eukaryotic species have well established pangenomes.

Copy number variation including gene deletion generates intraspecific gene content variation in nearly all species [11]. This variation can create extreme variance in fitness and promote adaptive evolution [12–15]. In plant pathogens, the ability to infect a host often relies on the secretion of effector proteins that interfere with the host cell machinery [16–18]. Host plants evolved cognate resistance proteins that are able to recognize effector proteins and trigger immunity [19]. Gains and losses of effector genes can therefore have a major impact on the outcome of host-pathogen interactions and challenge food security. Recent studies on fungal pathogens highlighted that genes showing presence-absence variation are enriched for predicted effectors [14, 20, 21]. Effectors and transposable elements (TEs) are often tightly associated with fast-evolving compartments of the genome [22, 23], also known as the “two-speed” genome architecture [24]. However, how TEs impact the birth and death of effectors in fast-evolving compartments remains largely unclear [6, 25]. The construction of pathogen pangenomes enabled crucial insights into functional diversity and the evolutionary trajectories of host adaptation. Recent pangenome analyses of four fungal species including opportunistic pathogens revealed that between ~ 9 and 19% of the pangenome is accessory. Accessory gene localization was preferentially in subtelomeric regions, suggesting both a mechanistic link to repeat-rich regions and relaxation of selective constraints [26]. The wheat pathogen *Zymoseptoria tritici* was found to have one of the largest eukaryotic pangenomes with an estimate of at least 42% of all genes being accessory [27]. However, eukaryotic pangenomes remain shallow and are often based on not fully resolved chromosomal sequences.

Fungal plant pathogens such as *Z. tritici* show extreme cases of genome plasticity. The reference genome of *Z. tritici* has 21 chromosomes, of which eight are accessory and segregate presence-absence variation in populations [28]. The pathogen rapidly evolved virulence on resistant wheat cultivars and has overcome all current fungicides [29–31]. Host adaptation was driven among other factors by the rapid deletion of an effector gene and structural rearrangements [32–34]. Pathogen populations are highly diverse with high rates of recombination [35–37]. Meiosis can trigger large chromosomal rearrangements and lead to aneuploid chromosomes in the species [38, 39]. A pangenome constructed for five *Z. tritici* isolates revealed that chromosome length variation segregating within populations was mainly due to the presence-absence variation of large TE clusters [27, 40]. Furthermore, accessory genes tended to form clusters dispersed along chromosomes. Accessory genes also tended to be in closer proximity to TEs than core genes and were therefore more likely to be affected by epigenetic silencing [27]. However, the constructed pangenome was very likely incomplete given the fact that four of the genomes originated from isolates collected in the same year from two nearby fields. Furthermore, accessory genes were enriched for pathogenesis-related functions but the pangenome size did not reach saturation. Given the global impact of the pathogen and the importance of accessory genes for adaptive evolution, a comprehensive pangenome capturing worldwide genetic diversity is essential.

In this study, we construct the pangenome of *Z. tritici* by including 19 isolates sampled from six different continents and covering the global distribution of the pathogen. We test to what extent the species segregates chromosomal rearrangements and how this impacts gene presence-absence variation at loci relevant for pathogenicity. We also analyse whether TE content is polymorphic within the species and may contribute to genome size evolution.

## Results

### Major chromosomal rearrangements segregating within the species

**Fig. 1:**
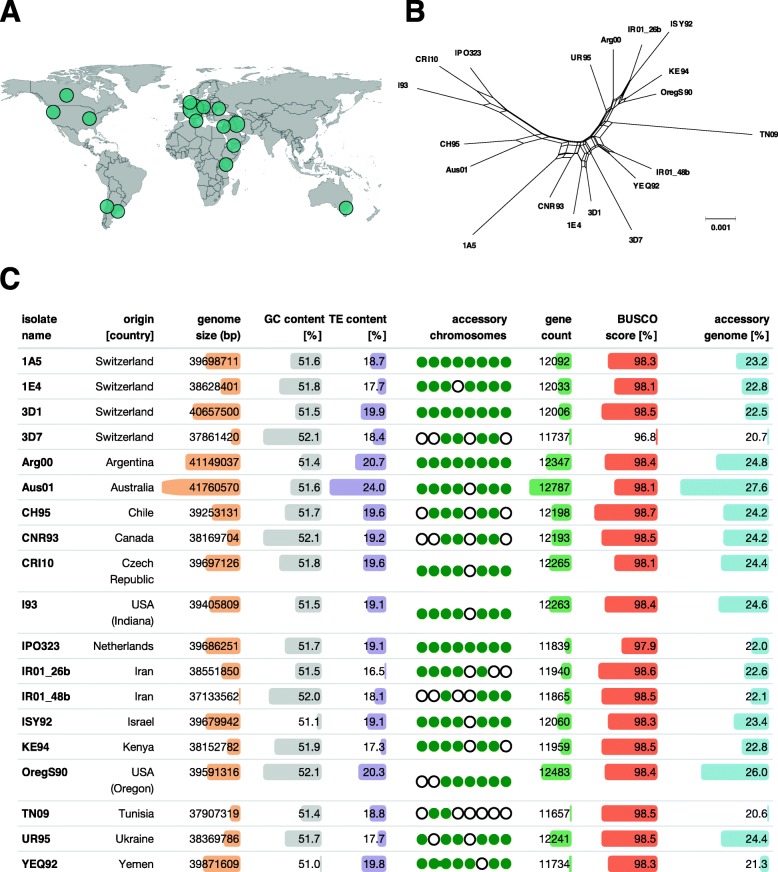
Assembly of 19 complete genomes from a worldwide collection. **a** World map indicating the isolate names and country of origin. **b** Phylogenomic tree based on 50 single-copy orthologs showing reticulation using SplitsTree. **c** Summary of genome assembly characteristics for all isolates. The bars represent the range of minimum (shortest bar) to maximum values (longest bar) for each reported statistic. Chromosome 14–21 are accessory chromosomes. The presence or absence of accessory chromosomes in each genome is shown by green dots and empty circles for present and missing chromosomes, respectively. The linked dots for isolate YEQ92 indicate the chromosomal fusion event (see also Fig. 2)

We constructed a global pangenome of *Z. tritici* based on 19 isolates sampled from six continents and 13 different countries (Fig. 1a, b). The isolates included the previously described reference isolate IPO323 sampled in the Netherlands and four isolates that were isolated from two nearby fields in Switzerland [27, 28, 40]. The geographic regions of origin of the 19 isolates recapitulate a significant environmental gradient in mean annual temperature and humidity and span the distribution range of the species. The sampling period ranges from 1984 (IPO323) to 2010 (CRI10). Fungicide applications against *Z. tritici* became widespread in the 1990s and early 2000s; hence, the sampling covers both pre- and post-fungicide treatment regimes. We sequenced long-read PacBio SMRTbell libraries to a depth of 40-110X and ~ 20 kb read coverage in order to generate chromosome-level assemblies. Assembly sizes ranged from 37.13 Mb (IR01_48b) to 41.76 Mb (Aus01) (Fig. 1c).

**Fig. 2:**
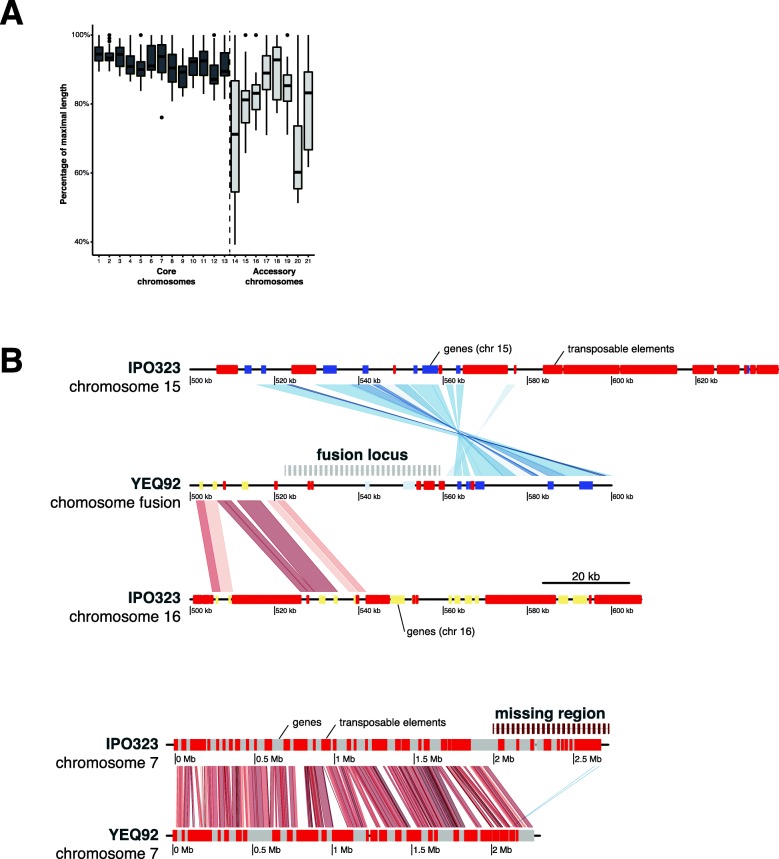
Large segregating chromosomal rearrangements within the species. **a** Chromosome length variation expressed as the percentage of the maximum observed length for each chromosome. **b** Two large chromosomal rearrangements identified in the isolate YEQ92 isolated from Yemen. The upper part shows the local chromosomal synteny at the fusion locus between accessory chromosomes 15 and 16 identified in YEQ92 compared to the reference genome IPO323. Transposons are shown in red, genes from chromosome 15 in purple, genes from chromosome 16 in green and genes specific to the fusion in grey boxes, respectively. Synteny shared between chromosomes is shown in red for colinear blocks or blue for inversions. The lower part shows the whole chromosome synteny of chromosome 7 contrasting YEQ92 to the reference genome IPO323. YEQ92 misses a subtelomeric region. Transposons are shown in red and genes in grey

We recovered all eight known accessory chromosomes of the species but no additional chromosome. The accessory chromosome 18 is most often missing. Together, the 8 accessory chromosomes display an average size variation of ~ 37% across all isolates and a maximum of 60% for chromosome 14 (Fig. 2a). For core chromosomes, the average size variation accounts for 16% of chromosome length going up to 23% for chromosome 7. We identified a major deletion spanning 406 kb and encompassing 107 genes on the right arm of core chromosome 7 of the Yemeni isolate (YEQ92; Fig. 2b lower panel). The same isolate had chromosome 15 fused to the right arm of chromosome 16. The fusion event is supported by aligned PacBio reads spanning the region between the two chromosomal segments (Additional file 1: Figure S1). The resulting chromosome is 1.20 Mb long and 49.5 kb shorter than the sum of the homologous chromosomes 15 and 16 of the IPO323 reference genome. Approximately 90% of the genes on the IPO323 chromosome 15 and 16 belong to accessory orthogroups, as they lack an ortholog in at least one of the other isolates. We find that the chromosomal fusion deleted about 150 kb affecting 1 and 12 genes on chromosomes 15 and 16, respectively (Fig. 2b upper panel). We further assessed genome completeness using BUSCO analyses. All genomes exceed the completeness of the fully finished IPO323 reference genome (97.9%) with the exception of isolate 3D7 (96.8%; Fig. 1c).

### Substantial gene content variation across the pangenome

**Fig. 3:**
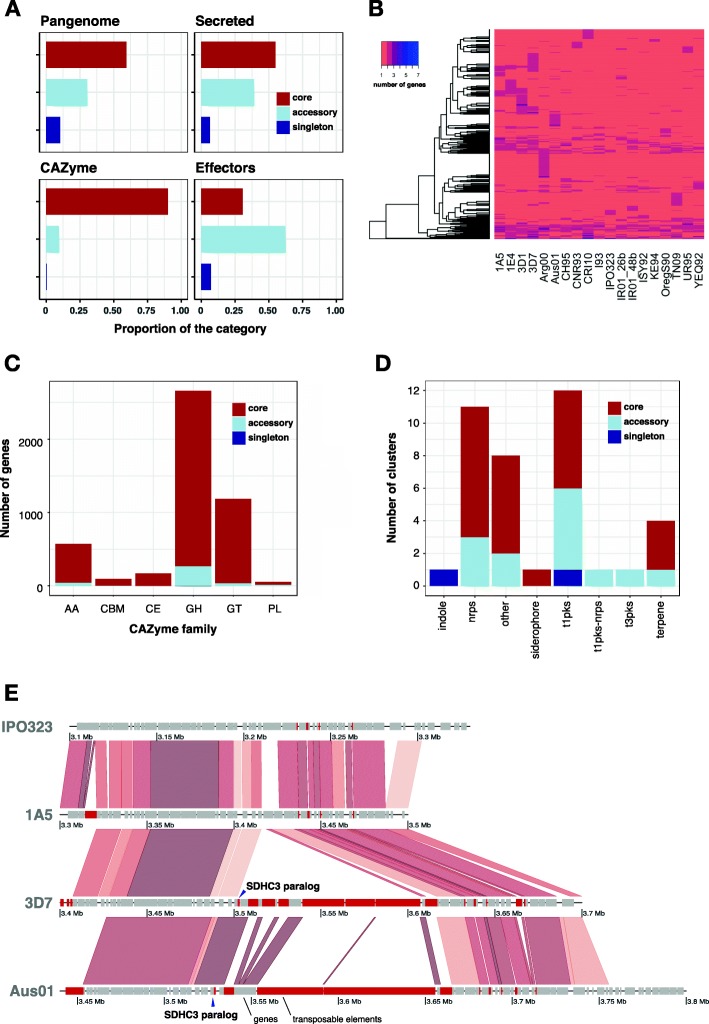
Construction and analysis of the *Zymoseptoria tritici* pangenome. **a** Proportions of core orthogroups (present in all isolates), accessory orthogroups (present ≥ 2 isolates but not all) and singletons (present in one isolate only) across the pangenome (upper-left). The proportions of core, accessory and singleton categories are shown for orthogroups coding for secreted proteins (upper-right), carbohydrate-active enzymes (CAZymes; lower-left) and effectors (lower-right). **b** Gene copy number variation in core orthogroups across the 19 genomes. **c** Pangenome gene count across six CAZyme families. Families are divided into glycoside hydrolase (GH), glycosyl transferase (GT), auxiliary activity (AA), carbohydrate esterase (CE), carbohydrate-binding modules (CBM) and polysaccharide lyase activity (PL) categories. **d** Pangenome categories of secondary metabolite gene clusters. **e** Synteny plot of succinate dehydrogenase (SDH) paralogs mediating fungicide resistance. The SDHC3 locus on chromosome 3 is shown for isolates 3D7 and Aus01 both carrying the paralog. IPO323 and 1A5 lack SDHC3. The position of the SDHC3 paralog is shown using dark arrows. Genes are coloured in grey and transposable elements in red

We generated RNAseq data to identify high-confidence gene models in all 14 newly assembled genomes based on a splice-site informed gene prediction pipeline. The total gene count varied between 11,657 and 12,787 gene models (Fig. 1c). We assigned all genes to orthogroups using protein homology and constructed a pangenome of all 19 complete genomes. The pangenome consists of a total of 229,699 genes assigned to 15,474 orthogroups. The number of genes assigned per orthogroup varies among isolates (Fig. 2b). Approximately 99.8% of all orthogroups (15,451) are single-gene orthogroups and ~ 60% of all orthogroups are shared among all 19 isolates (9193 core orthogroups). Around 96% of the core orthogroups (8829 out of 9193) have conserved gene copy numbers among isolates. Furthermore, we find that 30% of all orthogroups are shared between some but not all genomes (4690 accessory orthogroups) and 10% of the orthogroups are composed of genes found in a single genome only (1592 singletons; Fig. 3a, b; Additional file 2: Table S1).

To infect wheat, *Z. tritici* relies on specific gene functions [41, 42]. Effectors play a major role in establishing infection and exploiting host resources. Hence, we analysed how gene functions were structured across the pangenome components. Core orthogroups showing variation in gene-copy number among isolates include five encoding predicted effectors. Both accessory proteins and overall effector proteins are less conserved than core proteins at the amino acid level (Additional file 1: Figure S2). A total of 3.5% (691) of all orthogroups encode at least one predicted effector. Among orthogroups encoding at least one predicted effector, 31% were conserved among all isolates (219), 63% were accessory (436), and 5% were found in only one isolate (36 singletons). Notably, 99% of the predicted effector genes are located on core chromosomes. In addition to effectors, enzymes enabling access to nutrients are important pathogenicity components. We identified a total of 4742 annotated carbohydrate-degrading enzymes (CAZymes) clustered into 263 orthogroups. Notably, 92% of the orthogroups encoding CAZymes were conserved among all isolates (Fig. 3a). CAZymes grouped into 123 subfamilies. Glycoside hydrolases (GH) are the largest family and account for 57% of all annotated CAZymes (151 orthogroups for 2717 genes). Glycosyl transferases (GT) are the second most abundant family with 1188 genes and 66 orthogroups (25% of all CAZymes) (Fig. 3c). We also identified 33 orthogroups encoding for auxiliary activities (AA), 9 for carbohydrate esterase activity (CE), 6 for carbohydrate-binding modules (CBM) and 3 for polysaccharide lyase activity (PL). The PL family includes 29% accessory genes. Across CAZyme families, 0–10% of the genes are accessory (Fig. 3c). We found a singleton GH43 subfamily gene in the genome of the Australian isolate (Aus01).

The production of secondary metabolites contributes significantly to virulence and competitive abilities of fungal pathogens. We identified between 29 and 33 secondary metabolite gene clusters per genome depending on the isolate. A total of 70% of all genes predicted as components of a biosynthetic gene cluster are conserved between all isolates and 30% are accessory (Fig. 3d, Additional file 1: Figure S3). Of the 147 orthogroups annotated as encoding biosynthetic or biosynthetic-additional proteins in the pangenome, 87, 92, 111 and 112 have a homologue with > 50% identity in the four closely related sister species *Z. passerinii*, *Z. ardabiliae*, *Z. pseudotritici* and *Z. brevis*, respectively (Additional file 1: Figure S4). We identified 39 syntenic gene clusters in the pangenome classified into 12 type 1-polyketide synthase (PKS), 11 non-ribosomal peptide synthetase (NRPS), four terpene, one type 3-PKS, one siderophore, one indole and eight unclassified clusters. Sixteen (40%) of the identified syntenic clusters show presence-absence variation. In the CH95 isolate, a gene cluster on chromosome 7 was annotated as unclassified but annotated as a NRPS in 17 other isolates and absent from the IPO323 reference genome. The sole indole and type 1-PKS clusters located on chromosomes 5 and 10, respectively, were only found in isolate TN09. Two type 1-PKS and one NRPS cluster were missing in the isolates YEQ95, Aus01 and IPO323, respectively. Among the 39 identified syntenic gene clusters, 23 included a predicted effector and nine included a gene annotated as a cell-wall degrading enzyme.

The emergence of fungicide tolerance in *Z. tritici* is a major threat to wheat production. Succinate dehydrogenase (SDH) inhibitors are commonly used as control agents [31, 43]. We identified five SDH orthologs, of which three were conserved among all genomes (SDHB, SDHC and SDHD subunits). We find two distinct SDHC paralogs SDHC2 and SDHC3 in 11 and two isolates, respectively. The SDHC3 paralog conferring standing resistance to SDH inhibitors is located adjacent to a large cluster of TEs, suggesting that chromosomal rearrangements were underlying the paralog emergence (Fig. 3e). Genes encoding major facilitator superfamily (MFS) transporters, which can confer multidrug resistance in *Z. tritici* [44], grouped into 336 orthogroups for a total of 5787 genes (Additional file 2: Table S2). We find that 39 (11%) of these orthogroups are part of a predicted secondary metabolite gene cluster and one is an annotated CAZyme from the GH78 family. Overall, the results reveal that gene families essential for pathogenicity and fungicide resistance show unexpectedly high levels of presence-absence variation in the *Z. tritici* pangenome.

### Strong expression variation across major gene functions

**Fig. 4:**
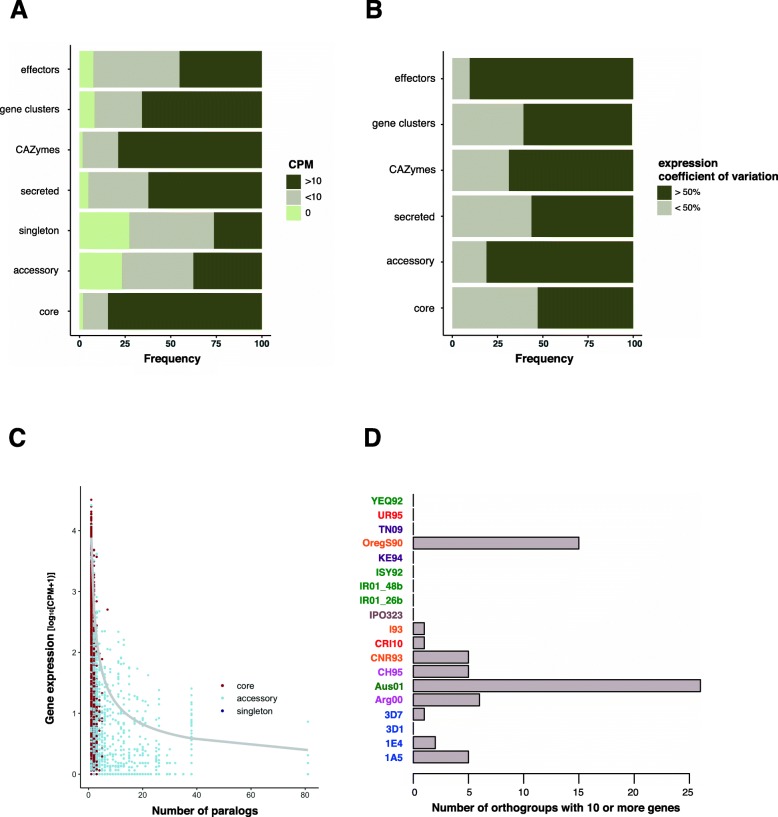
Expression polymorphism across the pangenome. **a** Proportion of genes showing expression > 10 counts per million (CPM) across genes categories. The frequencies are shown for orthogroups encoding putative effectors, secondary metabolite cluster genes (gene cluster), carbohydrate-active enzymes (CAZymes), secreted proteins. The frequencies are also shown for singleton, accessory and core orthogroup categories in the pangenome. **b** Proportion of orthogroups for which the expression coefficient of variation is > 50% [*cov* = *sd* (CPM)/*mean* (CPM)] among different gene and pangenome categories as in **a**. **c** Correlation of gene expression and the number of paralogs detected for the same gene per genome. The grey line shows the logarithmic regression based on the linear model *log*_*10*_ (CPM + 1) *~ log*_*10*_ (number of paralogs). **d** Number of orthogroups with ≥ 10 paralogs per genome. Isolates are coloured by continent of origin

Differential gene expression is a major driver of intraspecific phenotypic differences. We performed mRNA-sequencing of all 19 isolates grown on minimal media. Minimal media induces filamentous growth of *Z. tritici*, mimicking the morphology and nutrient starvation that occurs early during plant infection. We investigated isolate-specific gene expression by self-mapping RNAseq reads to each isolate’s genome assembly. Overall, 91.3% of the genes show expression on minimal media and 68% have expression of more than 10 counts per million (CPM) (Fig. 4a). Core genes have higher expression than accessory genes (Additional file 1: Figure S5). Among the genes showing no expression on minimal media, 501 are predicted effector genes (8% of predicted effectors), 93 are predicted CAZymes (2% of CAZymes) and 838 are members of a predicted gene cluster (10% of all gene cluster genes). CAZymes are overall highly expressed on minimal media (~ 77% with CPM > 10) when compared to effectors (~ 45% with CPM > 10) and gene cluster genes (~ 60% with CPM > 10) (Fig. 4a). About 53% of core single-copy orthogroups with non-zero expression have a coefficient of variation > 50% (Fig. 4b). Similarly, ~ 68% of CAZymes and ~ 60% of genes that are part of a secondary metabolite cluster have expression coefficient of variation > 50%. In contrast, about 90% of orthogroups encoding predicted effectors have a coefficient of variation > 50%, together with ~ 81% of accessory orthogroups.

To identify broad patterns in the pangenome expression landscape, we performed a clustering analysis of all core single-gene orthogroups. We find that expression clustering does not reflect the geographical origin or genetic distance with the exception of the four Swiss isolates (1A5, 1E4, 3D1 and 3D7; Additional file 1: Figure S6). We also analysed the impact of copy-number variation on average expression and find that single-copy orthologs are on average more highly expressed. In addition, we show that gene expression rapidly decreases if an orthogroup includes 2–8 paralogs (Fig. 4c).

### A highly variable transposable element content within the species

TEs are drivers of pathogen evolution by generating adaptive genetic variation. To identify genes with a potential role in the mobilization of TEs, we analysed large homology groups. Among the orthogroups with 10 or more paralogs, ~ 88% of the genes encode proteins without homology in databases, ~ 7% of the genes encode nucleic acid binding functions (GO:0003676), ~ 2% of the genes encode a retrotransposon nucleocapsid (GO:0000943) and ~ 1.5% of the genes encode a DNA integration domain (GO:0015074). Orthogroups with 10 or more paralogs are all accessory. For isolates sharing the same large orthogroups, we identified variability in the gene copy number within those orthogroups. Indeed, the isolates Aus01 and OregS90 have 26 and 16 orthogroups, respectively, with more than 10 assigned genes. The isolates I93 and Arg00 count between one and six orthogroups and nine other isolates have no orthogroups larger than ten genes (Fig. 4d). Altogether, these results suggest that large orthogroups (> 10 genes) essentially regroup genes that are encoded by TEs. Our data also indicates regional TE-driven genome expansions given the enlarged genome sizes in Australian and North American isolates.

**Fig. 5:**
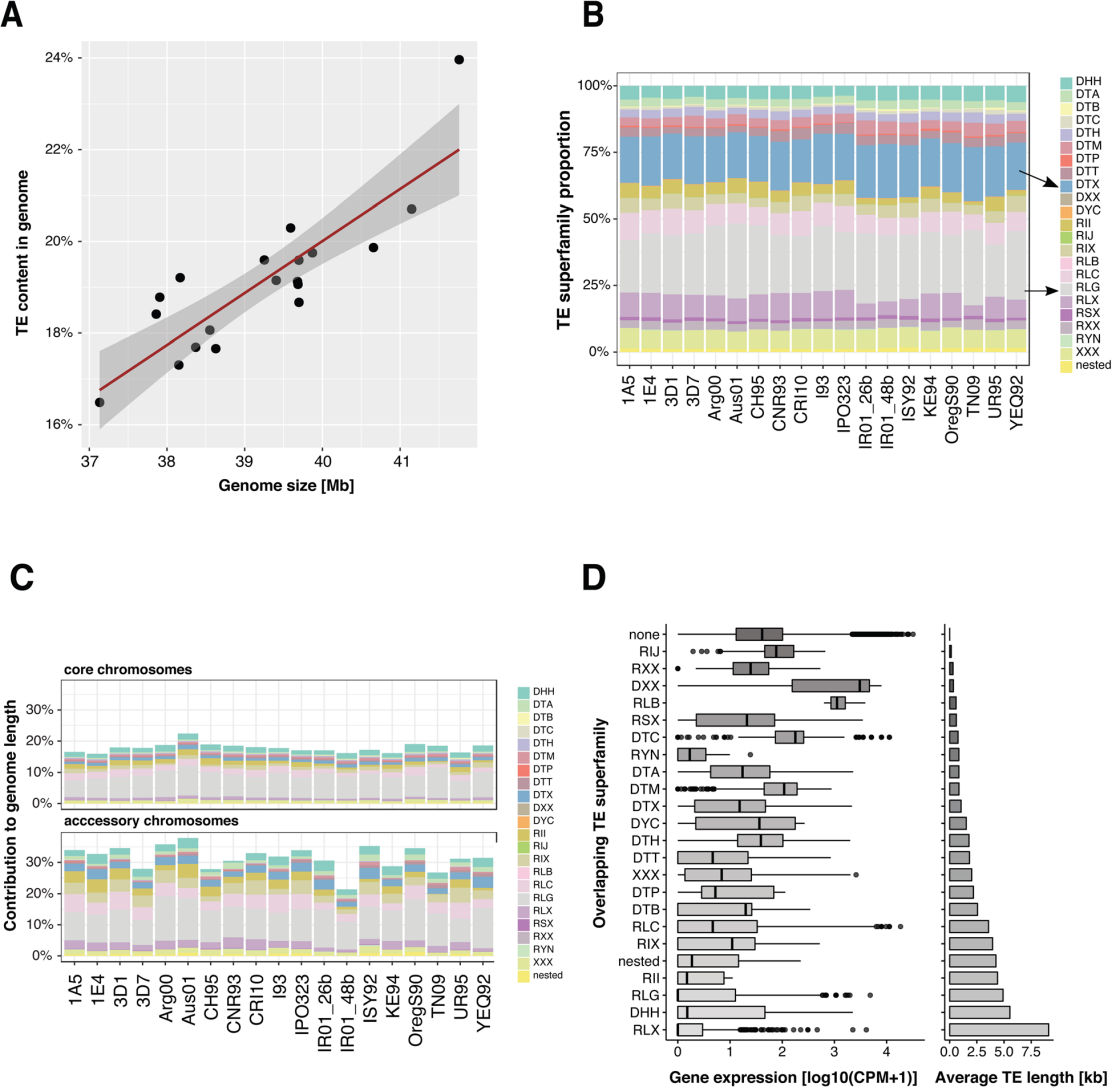
Transposable elements (TEs) and genome size variation. **a** Contribution of TEs (%) to total genome size across the 19 isolates. **b** Relative frequency of the 23 TE superfamilies across all genomes with 100% referring to the total TE content of the respective genome. **c** Contribution of TE superfamilies to core and accessory genome size across the 19 isolates. **d** Expression of genes affected by TE insertions (grouped by TE superfamilies; left panel) and the mean TE length in the genome (grouped by TE superfamilies; right panel)

To elucidate the role of transposition on generating genomic variation, we screened the 19 genomes for TE content. For this, we jointly analysed all complete genomes to exhaustively identify repetitive DNA sequences. We identified a total of 304 high-quality TE family consensus sequences grouped into 22 TE superfamilies. The GC content of the consensus sequences is highly variable, ranging from 23 to 77% (Additional file 1: Figure S7). On average, TE superfamilies have a GC content lower than 50%, except for unclassified SINE families (RSX; GC% ~ 50.6). The genomic TE content ranges from 16.48% (IR01_26b) to 23.96% (Aus01) and is positively correlated with genome size (cor = 0.78, *p* < 0.001; Fig. 5a). Genome size correlates with genome-wide TE proportions on both core and accessory chromosomes but is negatively correlated with the proportion of coding sequences (Additional file 1: Figure S8 and Figure S9). The average length of individual TEs ranges from 102 to 51,298 bp with the Helitron superfamily having the higher average length (Additional file 1: Figure S10-S11). The largest element is an unclassified LTR (RLX_LARD_Thrym) on chromosome 7, the size of which ranges from 6282 bp in CNR93 to 59,390 bp in ISY92. This particular LTR is present at the locus only in 18 isolates including ISY92, which has a fragmented secondary copy on chromosome 3. The RLX_LARD_Thrym insertion on chromosome 7 overlaps with the ribosomal DNA locus and showed far above average mapped PacBio read coverage (~ 250×).

The genome-wide content of TEs shows substantial variation among the 19 isolates; however, the relative abundance of different TE superfamilies is relatively conserved with LTR *Gypsy*, unclassified TIR and LTR *Copia* elements being the most frequent (Fig. 5b). Accessory chromosomes contain consistently higher proportions of TEs compared to core chromosomes (26–41% versus 17–24%; Fig. 5c). Aus01 and OregS90 isolates showed the highest TE content. Interestingly, the Aus01 genome shows LINE *I*, LTR *Gypsy* and LTR *Copia* family-specific expansion compared to other genomes. In contrast, the genome of OregS90 shows evidence for expansions of Helitron, LTR *Gypsy* and LTR *Copia* families. On average, 10% of all TEs overlap with genes. Overall, singleton and accessory genes tend to be closer to TEs and contain more often TE insertions than core genes (Additional file 1: Figure S12-S13). The isolates Aus01 and OregS90 have 12.8% and 12.4% of all TEs overlapping with genes, respectively. In addition, Aus01 and OregS90 isolates have 7.4% and 5.4% of all genes that overlap with TEs, respectively (Additional file 1: Figure S14). The composition of TEs inserted into genes reflects the overall TE composition in the genome, with more abundant TEs being more often inserted into genes (Additional file 1: Figure S15). TEs can carry their own regulatory sequences and are often epigenetically silenced by the host. We found that orthogroups comprising a gene within 100 bp distance of a TE show stronger expression variation (~ 62% of orthogroups with a coefficient of variation > 50%) compared to other orthogroups (~ 54% of orthogroups with a coefficient of variation > 50%) (Additional file 1: Figure S16-S17). We also found that different TE superfamilies have contrasting effects on gene expression, with longer TEs having more drastic effects (Fig. 5d). On average, genes with an inserted TE have lower expression levels (log10 CPM ~ 1.7-fold) and a higher coefficient of variation (log10 CPM ~ 2-fold) compared to genes without an inserted TE (Additional file 1: Figure S18).

### TE transcription correlates with relative frequency across isolates

**Fig. 6:**
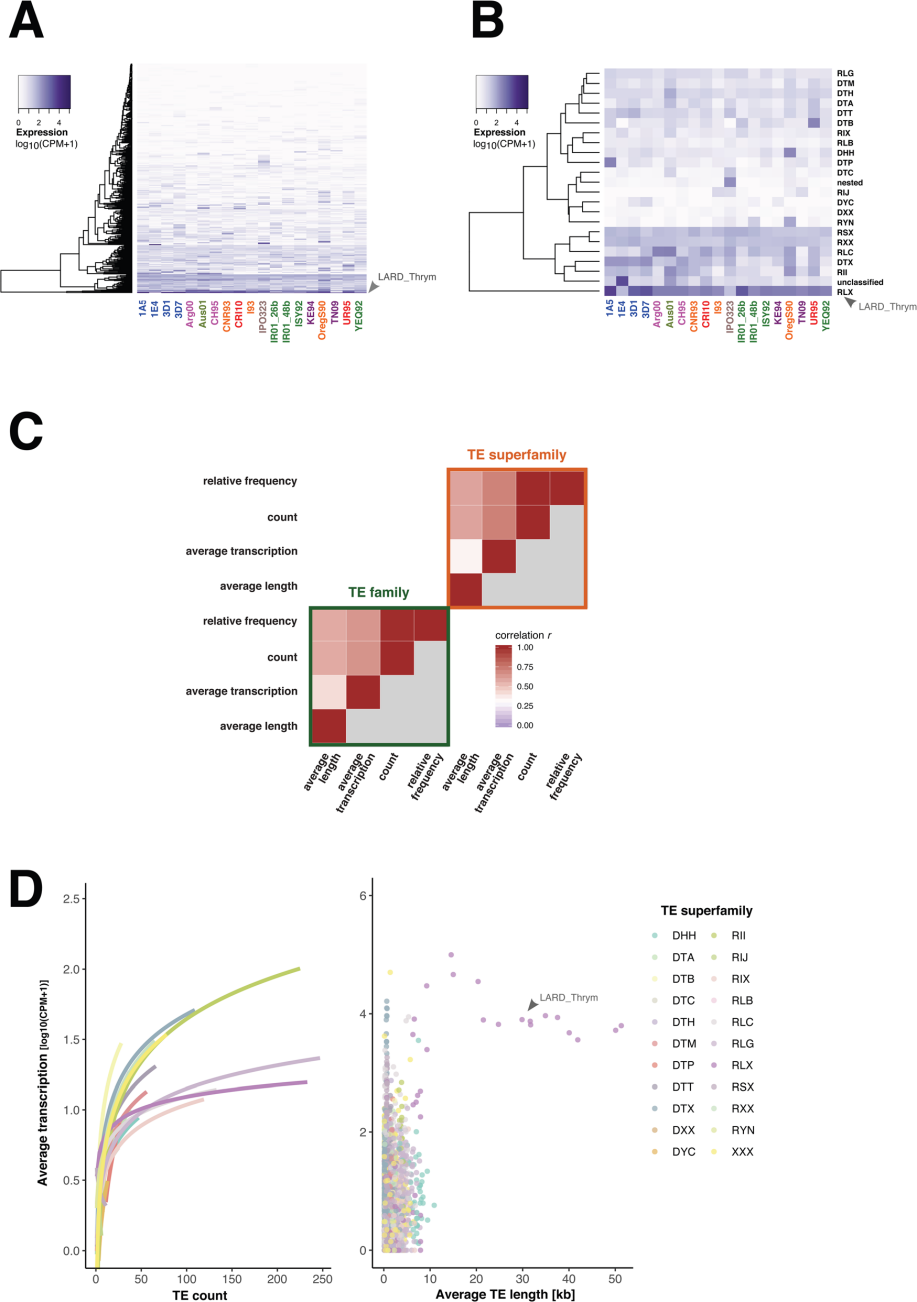
Transcriptional activity of transposable elements (TEs). **a** TE family transcription levels across all 19 genomes expressed as *log*_*10*_ (CPM + 1). **b** Average transcription levels of TE superfamilies across all genomes expressed as average *log*_*10*_ (CPM + 1). **c** Spearman correlation matrix of four TE metrics including counts, relative frequencies, average length and transcription both at the level of TE families and superfamilies. **d** Variation of TE transcription (average *log*_*10*_ (CPM + 1)) as a function of TE counts (left panel) or average TE length (right panel). Curves in the left panel show the logarithmic linear regression given by the linear model *log*_*10*_ (CPM + 1) *~ log*_*10*_ (TE count). The highly expressed LARD_Thrym family (RLX) is highlighted using arrows (panels **a**, **b** and **d**)

Class I TEs replicate through an RNA intermediate and class II through a DNA intermediate. Nevertheless, class II TEs can also transcribe into RNA. To gain insights into the mechanisms of proliferation, we analysed the relative abundance of TE-derived transcripts across all genomes. The highly repetitive nature of TEs typically prevents expression quantification at the individual copy level. Hence, we focused on normalized TE expression across all copies. Overall, more than 70% of the TE families have non-zero transcription levels. This is consistent with recent findings of pervasive transcription of TEs in the *Z. tritici* genome under nutrient stress and during infection [45]. We find that the largest TE family, an unclassified LTR identified as RLX_LARD_Thrym, was the most transcribed with an average log_10_ CPM ~ 4.2 (Fig. 6a). An unclassified DTX-MITE is the second most transcribed TE with an average log_10_ CPM ~ 3.6 followed by an unclassified TE (XXX_*Hermione* with an average log_10_ CPM ~ 3.4). At the superfamily level, LINEs have the highest expression overall followed by the aggregation of unclassified TEs (Fig. 6b). Retroelements are more transcribed than DNA transposons (average log_10_ CPM ~ 2 and 1.2, respectively).

To understand TE expression dynamics across the pangenome, we investigated associations between TE transcription, length and relative frequency (Fig. 6c). We found TE transcription to be correlated with TE frequency in the genomes (Spearman’s *r* = 0.49, *p* < 5e−307; Fig. 6c) and we found an even stronger correlation at the TE superfamily level (Spearman’s *r* = 0.59, *p* < 5e−40). However, TE transcription is not correlated with TE length at the superfamily level (Spearman’s *r* = 0.06, *p >* 2e−1; Fig. 6c). Interestingly, the average TE transcription levels are positively correlated with TE count in the genome (Fig. 6d). A notable exception is unclassified SINE retroelements. The correlation of TE transcription levels and TE frequency in the genome strongly suggests that transcriptional activity contributed to recent TE expansions in the genome.

## Discussion

We established a global pangenome of a major fungal wheat pathogen based on the assembly and analysis of 19 high-quality genomes. *Z. tritici* segregates major chromosomal rearrangements affecting both the more conserved core chromosomes as well as the highly polymorphic accessory chromosomes. The gene content is highly variable among genomes with only 60% of all genes being conserved in the species. Accessory genes encode functions for a wide variety of interactions with both biotic and abiotic environments. An exhaustive map of TEs across all genomes pinpoints transposon-associated genome expansions across geographic regions.

We showed that the *Z. tritici* pangenome is expansive with ~ 40% accessory orthogroups. Compared to a previous construction of the *Z. tritici* pangenome based on genomes from a much narrower geographic breadth [27], we used more relaxed criteria to assign genes into orthogroups. Based on the tendency to assign more divergent gene variants into the same orthogroup, we recovered a total of 911 orthogroups with at least one paralog compared to only 76 identified previously. The number of paralogs remains low compared to species with larger genomes that retained more paralogs of gene duplication events [28]. A likely constraint on gene duplication is the genomic defence mechanism that introduces repeat-induced point (RIP) mutations [46]. Although these defences evolved to suppress transpositional activity of TEs, they can also affect genome evolution by targeting gene duplicates [46, 47]. Recent sequencing efforts oriented around important crop species reported impressively large accessory genome proportions [48–50]. However, nearly all eukaryotic pangenomes are partially based on short-read assemblies that challenge the resolution of segregating gene variants within a species. With the conservative estimate of ~ 24% non-reference orthogroups, the *Z. tritici* accessory genome is the largest reported for a fungal species to date (~ 40% of the pangenome). This falls outside the upper range of comparative analyses of human fungal pathogens and *S. cerevisiae*, where estimates of the accessory genome ranged from 10 to 20% [26]. However, bacterial accessory genomes can range from 0 to 95% of the total pangenome [3]. The effective population size of a species, its lifestyle, and niche heterogeneity are main factors influencing bacterial pangenome sizes [4]. Similar to bacteria, the effective population size is likely to be the major factor maintaining a large accessory genome in *Z. tritici*. Previous studies identified *Z. tritici* as a highly polymorphic species with a rapid decay in linkage disequilibrium, high SNP densities and high recombination rates [32, 35]. As a consequence, the pathogen likely retains significant functional variation within populations as long as the variation is nearly neutral.

Bacterial and fungal genomes show clear functional compartmentalization between core and accessory genes [4, 26]. In fungi, core orthogroups are enriched for housekeeping functions in contrast to an enrichment for antimicrobial resistance and pathogenicity factors among accessory genes [27]. Here we show that genes encoding carbohydrate-active enzymes (CAZymes) are highly conserved within the species. CAZymes are involved in the degradation of the host cell wall and other storage compounds [51, 52]. Strong conservation of the content in CAZymes may reflect a fundamental adaptation to wheat as a host plant. This contrasts with generalist pathogens, which often evolved larger CAZyme repertoires [53]. In contrast to CAZymes, secondary metabolite gene clusters show substantial presence-absence variation within the species. Fungi produce highly diverse secondary metabolites that play a role during various life cycle stages, but often have poorly understood functions [54]. Plant pathogens were also shown to depend on secondary metabolite production for full virulence [55]. Hence, variation in secondary metabolite production may underlie variation in virulence. Species from the genus *Aspergillus* produce a large diversity of secondary metabolites for which the gene clusters often segregate presence-absence [56, 57]. The *Z. tritici* pangenome was constructed from isolates coming from six different continents and a wide array of agricultural environments. Hence, differences in secondary metabolite production capacity may reflect local adaptation and trade-offs that balance the cost of metabolite production. Virulence of *Z. tritici* is thought to be largely governed by gene-for-gene interactions [58]. In such interactions effector proteins either promote disease or are recognized by the host and trigger resistance [19]. A gene encoding a recognized effector should therefore be rapidly eliminated from the species gene pool. *Z. tritici* populations responded rapidly to selection on effector gene loci by either mutating, deleting or silencing genes [21, 33, 34]. Our global pangenome analysis significantly expands our understanding of effector gene diversification. We identified 652 orthogroups encoding predicted effector functions of which 63% are accessory orthogroups. Accessory effector genes may be involved in arms races with strong selection driving the gain or loss of individual effector genes in populations. As a contrast, we identified 45 conserved and highly expressed effectors genes potentially encoding indispensable pathogenicity functions.

Ultimate mechanisms promoting intra-specific diversity in genome structure may include large population sizes and niche complexity, however the proximate mechanisms generating such diversification are poorly understood. TEs can be key drivers generating structural variation [59, 60] and *Z. tritici* readily undergoes TE-mediated chromosomal rearrangements during meiosis [38, 39]. Here we show that *Z. tritici* genomes contain 16–24% TEs, with the overall proportion of TEs accounting for ~ 70% of the intraspecific genome size variation. Hence, TEs are key drivers of genome evolution in this species. Among the most drastic chromosomal rearrangements, we detected a significantly shorter chromosome 7 homologue. The longer homologue was hypothesized to have originated from a fusion with an accessory chromosome based on evidence from large-scale epigenetic remodeling [61]. Our analysis likely identified the ancestral variant prior to the suspected chromosomal fusion event. Hence, the species retained two major chromosomal variants of a core chromosome.

TEs are often implicated in gene copy number variation through duplication or pseudogenisation events suggesting that TEs directly contribute to pangenome diversification. We show that specific *Gypsy* and *Helitron* elements were integrated into genes generating highly paralogous orthogroups. These orthogroups may underlie recent expansions of specific TEs in the genomes of Australian and Oregon isolates. The *Helitron* element is among the most transcribed TEs in the Oregon isolate, suggesting a high potential for new transpositions. In contrast, the *Gypsy* element is only weakly transcribed in the Australian isolate, suggesting that this TE has become deactivated by genomic defences. In addition to transpositional activity causing loss-of-function mutations in genes, TEs can also contribute to genome expansions [62]. We found a strong correlation of TE content and genome size across the pangenome suggesting that TEs are the primary drivers of genome expansions. Because the pathogen was only recently introduced to regions outside of Europe and Asia, genome size variation among geographic regions may have originated from population bottlenecks such as founder events. As an example, populations in Australia underwent a significant founder event during the recent colonization of the continent from Europe [63]. Hence, our observation of an expanded Australian genome may be causally linked to this bottleneck. Genome expansions may also be triggered by TE mobilization. Stressors such as host defences during infection cause substantial TE de-repression across the *Z. tritici* genome [45]. Taken together, TE dynamics and large effective population sizes likely constitute the proximate and ultimate drivers of pangenome size evolution. Understanding the birth and death cycles of gene functions in such evolving pangenomes will help address major questions related to crop-pathogen co-evolution.

## Methods

### High molecular-weight DNA extraction and single molecule real-time (SMRT) sequencing

Origin and year of sampling of all the isolates are described in Additional file 2: Table S3. High-molecular-weight DNA was extracted from lyophilized spores following a modified version of a cetyltrimethylammonium bromide (CTAB) protocol developed for plant tissue described in [40]. Briefly, ~ 100 mg of lyophilized spores were crushed with a mortar and transferred to a phenol-chloroform-isoamyl alcohol solution. The supernatant was centrifuged and the pellet resuspended twice in fresh phenol-chloroform-isoamyl alcohol. The resulting pellet was then washed three times and resuspended in 100 μl of sterile water. For each isolate, PacBio SMRTbell libraries were prepared using between 15 μg and 30 μg of high molecular-weight DNA. Sequencing was performed on a PacBio Sequel instrument at the Functional Genomics Center, Zürich, Switzerland.

### Complete genome assemblies

We largely followed the pipeline described in [64]. In summary, raw PacBio sequencing reads were assembled using *Canu* v1.7.1 [65]. All assemblies were performed with an estimated genome size of 39.678 Mb (--genomeSize). Two corrected error rates (--correctedErrorRate 0.045 and 0.039) and minimal read length (--minReadLength 500 and 5000) parameters were tested, and the most contiguous chromosome-level assemblies were retained for further analysis based on reference alignment. The scaffolding was quality-controlled by inspecting genome-wide dot plots against previously assembled and validated genomes for reference. For each isolate, raw reads were aligned to the newly assembled genome using *pbalign* v0.3.1 from Pacific Biosciences suite (https://github.com/PacificBiosciences/pbalign) to inspect potential mis-assemblies. The assemblies were polished twice using PacBio reads mapped back to the new assembly using the software Arrow v2.2.2 from the Pacific Biosciences suite with default settings (https://github.com/PacificBiosciences/GenomicConsensus) and chromosome-level assemblies were performed using Ragout v2.1.1 and the IPO323 isolate as a reference [66].

### RNA extraction, library preparation, sequencing and quantification

For isolates 1A5, 1E4, 3D1 and 3D7, RNA sequencing experiments on minimal media were performed by [45, 67]. Raw reads were retrieved from the NCBI Short Read Archive accession number SRP077418. Similarly, the 15 additional fungal isolates (Additional file 2: Table S3) were grown in YSB media (10 g sucrose + 10 g yeast extract per litre) and then 10e5 cells were inoculated on liquid minimal media without a carbon source [68] for 7–10 days prior to extraction to reach identical growth stages as for the previous RNA sequencing experiments. RNA was extracted using a NucleoSpin® RNA Plant kit following the manufacturer’s instructions. Library preparation was carried out according to the Illumina TruSeq Stranded mRNA Library Prep protocol with unique indexes for each sample. Single-end 100-bp sequencing was performed on a HiSeq 4000 at the iGE3 platform in Geneva, Switzerland. RNA-seq reads were first filtered using Trimmomatic v0.38 [69] using the following parameters: ILLUMINACLIP:TruSeq3-SE.fa: 2:30:10 LEADING:10 TRAILING:10 SLIDINGWINDOW:5:10 MINLEN: 50, and then aligned to the corresponding genome assembly using STAR v2.6.0a [70] allowing for multiple read mapping (parameters set as --outFilterMultimapNmax 100 --winAnchorMultimapNmax 200 --outFilterMismatchNmax 3). We used HTSeq-count v0.11.2 [71] with -s reverse and -m union parameters to recover counts per feature (joint counting of reads in genes and TEs). We calculated normalized feature counts expressed as counts per million, which accounts for library size, using the EdgeR package v3.24.3 [72]. We restricted our analyses to features with a count per million > 1.

### Gene prediction and genome annotation

We used the gene prediction pipeline BRAKER v2.1 to predict genes in the 14 newly assembled genomes [73–80]. BRAKER combines coding sequence and intron hints based on the mapping of conserved protein sequences and introns identified in RNA-seq data, respectively. The above described RNA-seq datasets were joined with predicted protein sequences from the reference isolate IPO323 [28] and used to predict gene features and guide splice site mapping. RNA alignment files were generated with HISAT2 v2.1.0 using the --rna-strandness R option [81]. The resulting bam files were provided to BRAKER (--bam option) together with mapped IPO323 reference proteins (--prot_seq option) to generate gene predictions for each assembled genome using the --alternatives-from-evidence = false --prg = gth --etpmode --fungus parameters. Orthologous genes were identified using protein sequences from all 19 isolates and Orthofinder v2.1.2 with default parameters [82, 83].

### TE consensus identification, classification and annotation

To obtain consensus sequences for TE families, individual runs of RepeatModeler were performed on the 19 complete genomes in addition to the genome of *Z. pseudotritici* [84]. The classification was based on the GIRI Repbase using RepeatMasker [85, 86]. In order to finalize the classification of TE consensus sequences, we used WICKERsoft [87]. The 19 complete genomes were screened for copies of consensus sequences with blastn filtering for sequence identity of > 80% on > 80% of the length of the sequence [88]. Flanks of 300 bp were added and new multiple sequence alignments were performed using ClustalW [89]. Boundaries were visually inspected and trimmed if necessary. Consensus sequences were classified according to the presence and type of terminal repeats and homology of encoded proteins using hints from blastx on NCBI. Consensus sequences were renamed according to a three-letter classification system [90].

A second round of annotation was performed based on predicted protein sequences of TE superfamilies from other fungal species. Here again, the 19 complete genomes were screened for a protein sequence of each superfamily using tblastn. Blast hits were filtered for a minimal alignment size of 80 bp and sequence similarity > 35%. Flanks of 3000 bp or more both up- and downstream of the sequence were then added. Hits were pairwise compared with dotplots using dotter and grouped into families based on visual inspection [91]. Finally, multiple sequence alignments were performed with ClustalW to construct consensus sequences and the consensus sequences were renamed according to the three-letter system [90]. TE consensus sequences are available from https://github.com/crolllab/datasets.

A third round of annotation of the 19 complete genomes was done to identify four groups of short non-autonomous TEs. LTR-Finder was used to screen for LARDs (LArge Retrotransposon Derivates) and TRIMs (Terminal Repeat retrotransposons In Miniature) with the filters -d 2001 -D 6000 -l 30 -L 5000 and -d 30 -D 2000 -l 30 -L 500 respectively. MITE-Tracker was used to screen for MITEs (Miniature Inverted-repeat Transposable Elements) and SINE-Finder in Sine-Scan to screen for SINEs (Short Interspersed Nuclear Elements) [92–97]. For each detected LARD, TRIM and SINE, consensus sequences were created as described above and duplicates excluded. All genome assemblies were then annotated with the curated consensus sequences using RepeatMasker with a cut-off value of 250 and ignored simple repeats as well as low complexity regions. Annotated elements shorter than 100 bp were filtered out, and adjacent identical TEs overlapping by more than 100 bp were merged. Different TE families overlapping by more than 100 bp were considered as *nested* insertions and were renamed accordingly. Identical elements separated by less than 200 bp indicative of putative interrupted elements were grouped into a single element using minimal start and maximal stop positions. TEs overlapping ≥ 1 bp with genes were recovered using the *bedtools* v2.27.1 suite and the *overlap* function [98]. Correlations were calculated in RStudio version 1.1.453 using Spearman’s coefficient for pairwise complete observations and statistics were inferred with the *psych* package using the Holm correction method [99].

### Functional annotation of predicted genes

Protein functions were predicted for all gene models using InterProScan v 5.31-70.0 [100] adding -goterms -iprlookup and -pathway information. Secretion peptides and transmembrane domains (TM) were identified using SignalP v 4.1 and Phobius [101, 102]. The secretome was defined as the set of proteins with a signal peptide but no TM as predicted by either SignalP and Phobius. Putative effectors were identified among the set of secreted proteins using EffectorP v 2.0 [103]. Carbohydrate-active enzymes (CAZymes) were identified using dbCAN2 release 7.0 server [104, 105] with the three tools HMMER, DIAMOND and Hotpep [106–108]. Proteins were classified as a CAZyme if predicted by each of the three tools. We searched for secondary metabolite gene clusters using the online version 4 of antiSMASH [109]. Genes belonging to an identified cluster were annotated as “biosynthetic”, “biosynthetic-additional”, “transport”, “regulatory” or “other”. Gene clusters mapping at a conserved, orthologous locus shared by two or more isolate were considered as syntenic.

## Supplementary information

Additional file 1: **Figure S1.** Integrative Genomics Viewer screenshot of PacBio reads aligned back to the YEQ92 genome assembly at the fusion locus between chromosomes 15 and 16. **Figure S2.** Percent identity given by the multiple protein sequence alignment for each orthogroup. Protein sequences were aligned using *mafft* and alignment identity was extracted with the *easel alistat* tool from Eddy Rivas (https://github.com/EddyRivasLab/easel). **Figure S3.** Presence-absence heatmap of the orthogroups assigned to secondary metabolite gene clusters. Each line stands for an orthogroup. Syntenic gene clusters are numbered from 1 to 39. Orthogroups including more than one gene per cluster are shown in darker blue (0–3 genes were found assigned to an orthogroup in this analysis). **Figure S4.** Evolutionary origins of the secondary metabolite genes clusters. We performed *blast* searches using all annotated biosynthetic and biosynthetic-additional proteins as query against four closely related sister species of *Zymoseptoria tritici*. The heatmap shows the percent identity of the top hit found in the four sister species for each of the 147 genes encoding biosynthetic functions in putative gene clusters. The isolates Zpa63, Zp13, Zb87 and Za17 correspond to the species *Z. passerinii*, *Z. pseudotritici*, *Z. brevis* and *Z. ardabiliae* respectively. **Figure S5.** Gene expression across pangenome categories. Gene expression is shown as log10 values of counts per million reads + 1 because non-expressed genes are also shown. **Figure S6.** Single-gene core orthogroups heatmap following hierarchical clustering based on Euclidian distances. Gene expression is shown as the log10 values of counts per million reads + 1 as non-expressed genes are also shown. **Figure S7.** GC-content across transposable element family consensus sequences. **Figure S8.** Transposable element (TE) content correlated with genome length for both core and accessory chromosomes. The proportion of TEs was calculated as the percentage of chromosome length in bp. **Figure S9.** The proportion of the genome covered by genes correlated with total genome size. **Figure S10.** Heatmap of average transposable element size (*log10* of the average length in bp). **Figure S11.** Heatmap of average transposable element size summarized by superfamily (*log10* of the average TE superfamily length in bp). **Figure S12.** Distance to closest transposable element across pangenome categories given as log_10_ values of the distance in base pairs. **Figure S13.** Proportion of pangenome categories overlapping with transposable elements (TE). All features with at least 1 bp overlap with a TE sequence were considered. **Figure S14.** Proportion of overlapping genes in blue and transposable elements (TE) in grey. All features with at least 1 bp overlap with a TE sequence were considered. **Figure S15.** Genome-wide transposable element (TE) superfamily frequencies correlated with the proportion of TEs overlapping genes. Proportions are given for each TE superfamily (colour code) and each of the 19 isolates. **Figure S16.** Frequency of orthogroups showing high (> 50%) and low (< 50%) expression coefficient of variation. Only orthogroups were distinguished whether at least one gene of the orthogroup was located within 100 bp of a transposable element or not. **Figure S17.** Gene expression as a function of its distance to the closest transposable element (TE). The relationship is shown for each of the TE superfamilies across the 19 isolates. Gene expression is given by the log10 values of normalized counts per millions reads + 1 as genes showing zero expression are also included. **Figure S18.** Gene expression of genes overlapping at least one base pair with a transposable element (“yes”) compared to genes not overlapping (“no”). Gene expression is given by *log10* values of counts per million reads.

Additional file 2: **Table S1.** List of all identified orthogroups and pangenome categorization. Table S2. List of the genes encoding Major Facilitator Superfamily domains (IPR036259). Table S3. Summary table of the analyzed isolates.

## Data Availability

The genome assembly and annotation for new genome assemblies are available at the European Nucleotide Archive (http://www.ebi.ac.uk/ena) under the BioProject PRJEB33986 [110]. The RNA-sequencing raw sequencing data was deposited at the NCBI Short Read Archive under the accession number PRJNA559981 [111].
